# Field-Dependent Rheological Properties of Magnetorheological Elastomer with Fountain-Like Particle Chain Alignment

**DOI:** 10.3390/mi13040492

**Published:** 2022-03-22

**Authors:** Muhammad Akif Muhammad Fakhree, Nur Azmah Nordin, Nurhazimah Nazmi, Saiful Amri Mazlan, Siti Aishah Abdul Aziz, Ubaidillah Ubaidillah, Fauzan Ahmad, Seung-Bok Choi

**Affiliations:** 1 Engineering Materials and Structures (eMast) iKohza, Malaysian-Japan International Institute of Technology, Universiti Teknologi Malaysia, Jalan Sultan Yahya Petra, Kuala Lumpur 54100, Malaysia; akif.fakhree@gmail.com (M.A.M.F.); nurhazimah@utm.my (N.N.); amri.kl@utm.my (S.A.M.); aishah118@gmail.com (S.A.A.A.); 2 Institute for Vehicle Systems and Engineering (IVeSE), Sultan Ibrahim Chancellery Building, Universiti Teknologi Malaysia, Jalan Iman, Skudai 81310, Malaysia; 3 Mechanical Engineering Department, Universitas Sebelas Maret, J1. Ir. Sutami 36A, Kentigan, Sukarta 57126, Indonesia; 4 Vehicle System Engineering (VSE) iKohza, Malaysian-Japan International Institute of Technology, Universiti Teknologi Malaysia, Jalan Sultan Yahya Petra, Kuala Lumpur 54100, Malaysia; fauzan.kl@utm.my; 5 Department of Mechanical Engineering, The State University of New York, Korea (SUNY Korea), 119 Songdo Moonhwa-Ro, Yeonsu-Gu, Incheon 21985, Korea; 6 Department of Mechanical Engineering, Industrial University of Ho Chi Minh City (IUH), 12 Nguyen Van Bao Street, Go Vap District, Ho Chi Minh City 70000, Vietnam

**Keywords:** magnetorheological elastomer, fountain-like alignment, storage modulus, MR effect, shear stress direction

## Abstract

Magnetorheological elastomer (MRE) consists of magnetic particles known as carbonyl iron (CIPs), which have been locked in a silicone-based matrix, in various alignments. However, current MRE exhibits inadequate rheological properties due to several issues such as particle alignments. Therefore, in this study, a new approach of the particle alignment of CIPs in MRE, namely fountain-like structure, is introduced. It is expected that this kind of MRE exhibits enhancement rheological responses, in off- and on-state conditions. This work includes the development of a new mold that can produce various directions of magnetic flux lines in order to have fountain-like structures of CIPs in MRE, during the curing process. Three types of particle alignments in MRE, namely isotropic, fountain-like and inverted fountain-like, are fabricated. The rheological properties of MRE in terms of storage modulus and MR effect are measured in an oscillatory shear mode using a rheometer. The results have revealed that fountain-like MRE exhibits higher storage modulus than the isotropic MRE, approximately 0.6 MPa of increment in the strain sweep test, in an on-state condition. Furthermore, it has been demonstrated from strain, frequency and the current sweep test that the rheological properties of fountain-like MRE related to storage modulus and magnetorheological (MR) effect are higher compared to the inverted fountain-like MRE.

## 1. Introduction

Magnetorheological elastomer (MRE) is a polymer composite that consists of magnetically permeable particles distributed within a non-magnetic elastomeric matrix [1,2]. MRE possesses rheological properties and offers variable stiffness, which can be controlled under the influence of an external magnetic field. The properties are attributed to the locked magnetic particles in the elastomer matrix that operatively responds to the applied magnetic field. The behavior of fast responsiveness and changeability of its stiffness has rendered MRE belongs to a group called smart material, particularly magnetorheological (MR) materials [3,4,5,6]. Possessing such advantages, MRE has created wider application opportunities including semi-active vibration dampers, vibration isolators, actuators and sensors [7,8,9,10]. In the presence of the magnetic field, changes in the viscoelastic properties of MRE are typically described by the MR effect [3]. On the other hand, the effect is a behavior that defines the changes of the storage modulus of MRE in response to the tunable magnetic fields against a set of specified strains [11,12]. The MR effect of MRE is comparable depending on the composition of magnetic particles and matrix components, types of matrices, concentrations and sizes of magnetic particles, additives and types of curing process that simultaneously affect the resultant viscoelastic properties of MRE [13,14,15,16,17,18].

Two types of MRE are characterized by the way magnetic particles are dispersed in the elastomeric matrix. The first dispersion is called isotropic MRE, which can be identified by a uniform distribution of particles in an MRE. This kind of MRE can be acquired by curing the melt MRE in a mold (mould) without applying a magnetic field, thus the particles will be uniformly dispersed in the cured matrix phase. Meanwhile, the second type of MRE is called anisotropic, represented by the aligned magnetic particles at a specific degree, in the MRE. The magnetic field that is applied during the pre-structure or crosslinking process of MRE will allow the particles inside the rubber matrix to align in a columnar configuration, forming chain-like structures according to the lines of magnetic field [19,20,21]. Generally, anisotropic MREs possess a higher MR effect and wider magneto-induced modulus compared to isotropic MRE [11,12,18,22,23]. This is due to a smaller gap between the inter-particles that are arranged in an aligned manner, resulting in the applied magnetic flux to easily flow along the aligned particles in the anisotropic MRE [24,25]. This concept will give rise to the MRE to highly respond towards the applied magnetic field. The closer gap between the particles will also offer a higher permeability for the magnetic flux to flow within the elastomeric matrix of the MRE [25,26]. On the other hand, in the presence of magnetic field, the aligned particles in an MRE are magnetically at the lowest energy state, making the attraction forces between the particles are at maximum strength [11,12,27,28,29]. This phenomenon in return has enhanced the capability of MRE to resist deformation when a shear force is applied onto it, or known as stiffness and, reasonably, the storage modulus as well as the MR effect of the anisotropic MRE increase. In fact, the interaction forces between the magnetizable particles in the aligned structure have resisted more deformation when the MRE sample was magnetized and sheared [13,20,30,31]. 

Furthermore, the study by Yao et al. [13] also paid particular attention to the magneto-induced modulus of the anisotropic MRE in which the corresponding behaviour could be improved by changing the orientation angle between the particle chains, respective to the applied magnetic field. The result demonstrated that the highest magneto-induced MRE was achieved at 30° particles chain’s angle, with the use of bigger particle sizes. Despite that, for the smaller size of the particles (below than 10μm), the magneto-induced modulus of MRE was noted higher at 45°. Another study was done by Boczkowska et al. [22] focusing on the polyurethane-based MREs that were fabricated with different angles of particles chain alignments. The result reported that the samples with 30° of particles chain alignment to the applied magnetic field (*y*-axis) exhibited the highest storage modulus compared to samples with 0°, 45° and 90°. The finding also illustrated that the magneto-induced modulus as well as the MR effect of MREs could be enhanced and manipulated through different particles’ chain alignments. Nevertheless, these findings have shown that the particles’ chain alignments have affected the resultant properties of the modified MRE. In another study, Tian et al. [32] who focused on the viscoelasticity properties of MRE with 45° of iron particle’s alignment stated that the movement of the rheometer plate (shearing mode) could stretch the particle chains or vice versa, affecting the resultant storage and loss moduli of the material. In fact, both storage and loss moduli of MRE were achieved higher when the applied shear stress direction crammed the particle chains as compared to the shear direction that stretched the particle chains. It was stated that, under the applied shear stress, the particle chains that were crammed along the shear direction would create more restraints on the matrix phase as the movement of the molecular chains was hindered by a higher density of the particles. This finding also corresponded to the previous study who stated in a theoretical modelling that, as the average distance between the sheared particles reduced, the resultant shear stress as well as the storage modulus of MRE would be increased [33].

Despite many investigations to discover the most significant orientation of particles that results in high performance of MRE, the inconsistency of shearing force distributed in the MRE during the oscillatory shear mode test should be highlighted as well. The homogeneity in shearing force distribution is believed to be an important factor to acquire maximum impact of the rheological properties of MRE, by considering both the orientations of the particle’s chain and the shear force direction. Zhang et al. [34] have shown the relationship between the orientation of particles-chains and shear force directions. Prior to the investigation, the sample was cut and repeatedly positioned in a rheometer symmetrically to ensure that the angle between the particle chain and shear path direction were in-line considering the shear path of a circular direction. The sliced technique of the study highlighted that both parameters—particles’ alignments and shear force directions—could be integrated by the oscillating plates and, as a result, the MRE produced a consistency value of storage modulus. The study somehow showed the importance of having various particle–chain alignments in an MRE that could face the change directions of applied shear force in order to produce maximum and consistent value of a storage modulus. 

Nevertheless, it is difficult to address which angle of aligned particles in MRE would result in the highest rheological effect since different angles are highlighted to achieve the improvement target. Therefore, in this current study, a new particle–chain alignment, called fountain-like particle chains in MRE, is introduced to accommodate various angles of particle alignments, especially in the rotation direction where the force is applied almost perpendicular to the CIPs alignment. Table 1 illustrates the example types of MREs with respective different alignments of CIPs, including the proposed fountain-like structure. The study also focuses on the correlation between the particle’s alignments and shear force directions in oscillation mode. Therefore, the study offers fundamental knowledge in designing MR devices, especially focusing on the appropriate particle’s alignment for a specific application. This approach has the potential to be applied for the in-situ fabrication of MRE devices where the particles will be cured and aligned following the direction of the magnetic field during the production process of the device. In fact, prior to the in-situ fabrication, the interactions between the CIPs and magnetic fluxes upon exact application of the device would be further strengthened.

## 2. Methodology

### 2.1. Preparation of MRE

The MRE matrix was prepared by using silicone rubber from RTV-two NS625 purchased from Nippon Steel, Tokyo, Japan. Meanwhile, the magnetic particles used were carbonyl iron particles (CIPs), supplied by CK Materials Lab Co., Ltd., Seoul, South Korea, with an average particle size of 3–6 μm. In addition, 30 wt.% of liquid silicone rubber was mixed with 70 wt.% of CIPs. Furthermore, 70 wt.% CIP was chosen for the sample because it is the best possible percentage with a stable response to magnetic stimulation and a higher MR effect [11,35,36]. The mixture of liquid silicone rubber and CIP were stirred by using a mechanical stirrer at a speed of 200 rpm, for 15 min. Then, the peroxide curing agent was added to the mixture and stirred for another 1 min. The melted mixture was then poured into a non-magnetic mold having a dimension of 1 mm thickness and 30 mm in diameter. Three groups of MRE samples were prepared at room temperature of 25 °C, with different particle chain alignment as shown in Table 1. The first sample, namely isotropic MRE, was cured in the absence of a magnetic field, indicating the randomness distribution of the CIPs in the MRE. Meanwhile, the second sample was cured under the influence of magnetic flux density of 0.2 T, which resulted in having various particle-chain orientations, namely fountain-like particles’ chain alignment. All samples were cured for two hours. From the second sample, the testing was carried out on two sides: “Fountain-like” and “Inverted Fountain-like” as stated in Table 2. 

### 2.2. Design of Mold 

**Figure 1:**
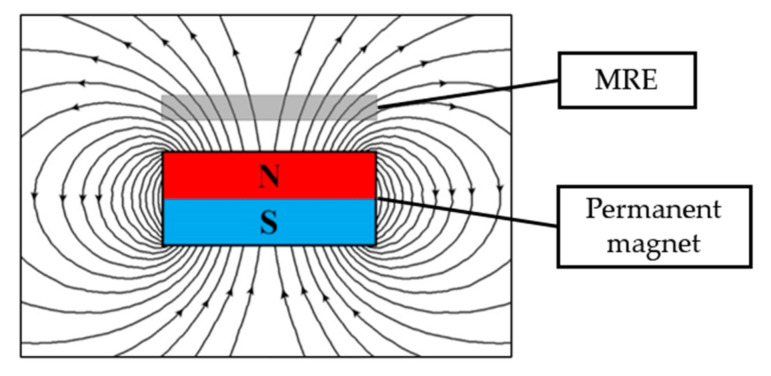
The schematic diagram of MRE for which induced magnetic flux densities would drive the magnetic particles (CIPs) in the MRE to align along the magnetic lines during curing.

The important parameter in producing the diverged, fountain-like particle chain alignments inside the MRE was by controlling the direction of magnetic flux density that penetrated in the samples during the curing process. Figure 1 shows the concept of MRE’s placement in a mold with induced magnetic flux density to the sample during the curing process. The figure illustrates the position of MRE in which the lines of magnetic flux densities would be a guidance for the CIPs to align during the pre-structuring process and the concept would be dependent on the strength of magnetic flux density that passed through the uncured MRE. The particles that would be rearranged along the directions of magnetic flux density was attributed to the magnetic interactions between the particles. Hence, the strength of the magnetic field was one of the major requirements in fabricating the anisotropic MREs as the alignment of magnetic particles in the silicone matrix would depend on the magnetic field strength [37].

In this study, an electromagnetic coil was used in the mold set up in order to create a controllable magnetic flux that passed through the sample. The coil for the apparatus was made from insulated copper wire and was wound to a plastic bobbin by about 1250 turns. Then, the disc-shaped magnet with a magnetic flux density of 0.36 T was placed in the center of the coil. The magnet that has a dimension of 30 mm in diameter and 8 mm in thickness was placed in the slot over the mold. The role of the disc-shaped permanent magnet was to capture the magnetic flux induced by the electromagnetic coil and to produce a curved flux line that is looping from its north pole to the south pole that passed through the uncured sample. Each flux line from the north pole has a definite direction to the south pole forming an individual closed loop. Both the utilization of electromagnetic coil along with the permanent magnet were to control the production of magnetic flux density across the mold. The apparatus was first simulated using a magnetostatics analysis software (FEMM); then, the output data were validated by using Gauss meter after the apparatus was developed. Details of the components and materials used during the fabrication are listed in Table 3.

**Figure 2:**
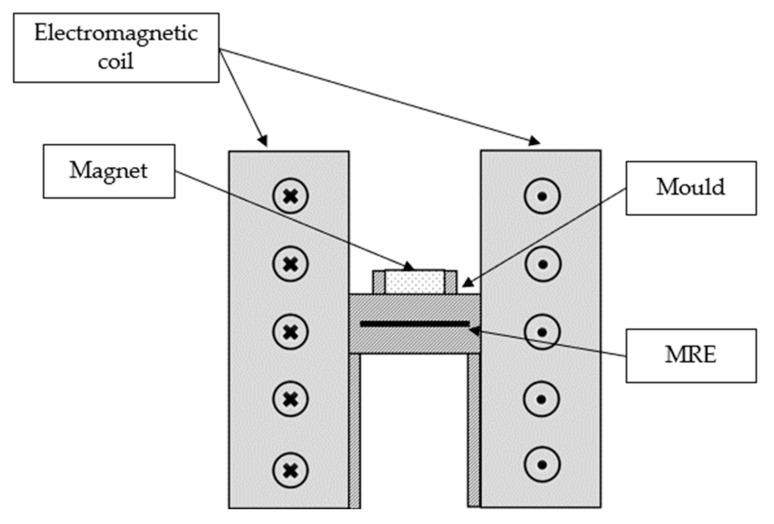
Schematic diagram of the electromagnetic coil set up.

**Figure 3:**
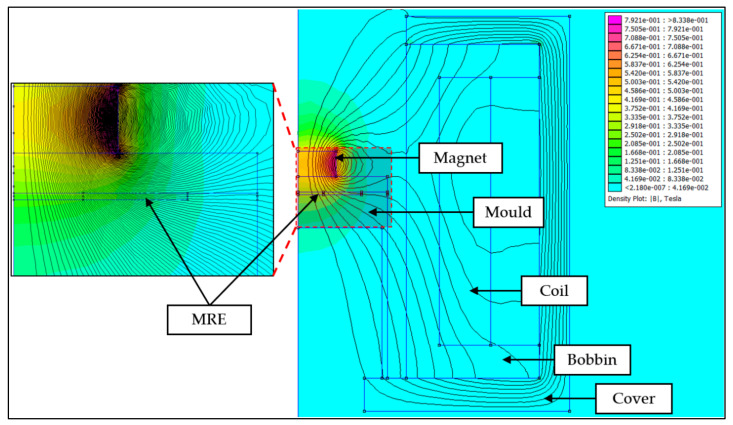
Simulation through FEMM showing the flux line generated from the apparatus.

Based on the design, the magnetic flux and lines produced were studied through finite element analysis by utilizing open-source software FEMM 4.2. The illustrations of the magnetic flux flow within the set-up coil were depicted in Figure 2. The simulation was conducted in a 2D axis-symmetry mode which showed half of the cross-sectional area that would actually represent the whole set-up system. In the FEMM software, each material with its magnetic properties was arranged accordingly as in the simulation. The non-magnetic mold was sandwiched between the metal cylinders to allow greater magnetic flux density to flow throughout the mold. The current was set at 1 A to produce minimum 0.2 T of magnetic flux density that would cross the mold, and to drive the CIPs to align within the matrix of MRE. Figure 3 shows the prediction of magnetic field and magnetic flux density generated within the set-up apparatus and its distribution in the uncured MRE.

**Figure 4:**
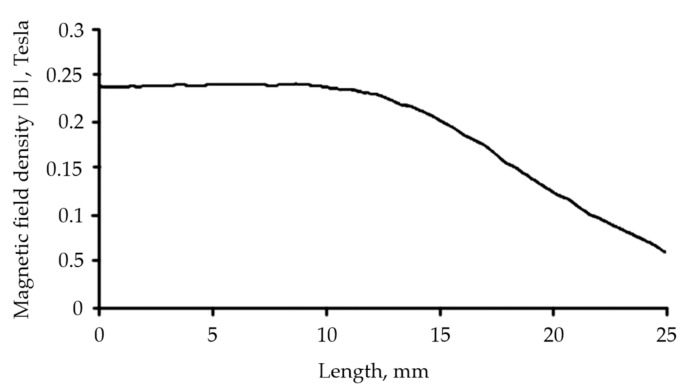
Magnetic flux density across MRE in the mold.

Meanwhile, the MRE with a fountain-like arrangement was predicted by adding a disc-shaped permanent magnet over the mold. The adding of the permanent magnet as a ‘pick-up’ component was to pick up the generated magnetic force by the magnetic coil; thus, it would produce curved magnetic lines as in Figure 1. The curved magnetic flux lines also would lead to the CIP alignments in the MRE. In fact, Figure 4 shows the results of simulation, indicating the amount of magnetic flux density that passed through the sample during the curing process. The results of the aligned particles then would be validated via morphological observation.

### 2.3. Characterization and Testing of Samples

#### 2.3.1. Morphological Characterization

**Figure 5:**
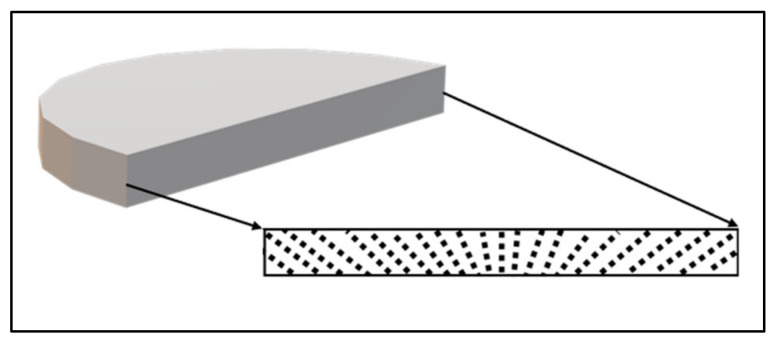
Cross-sectional surface of MRE for FESEM analysis.

The morphological characterization of the MRE samples was observed using a field emission scanning electron microscope (FESEM), SU8020 model from Hitachi High-Tech, Tokyo, Japan, with a magnification of 500× and 1000× at a voltage of 5 kV. The observation morphology was carried on the surface of the cross-sectional area of the MRE as demonstrated in Figure 5. The figure also illustrates the expected fountain-like alignment of the CIPs in the MRE.

#### 2.3.2. Rheological Test

The rheological properties of different samples were measured by using a rheometer (Physica MCR 302) from Anton Paar Co., Graz, Austria. The measurements were performed by sandwiching the MRE samples in a gap of 1 mm between the upper plate and the bottom plate of the sample holder. The upper plate as the rotor is the part of the measuring geometry, while the bottom plate is the stationary part that is mounted onto the rheometer stand. The dimensions of the samples were 20 mm in diameter and 1 mm in thickness. The tests were carried out under the oscillating shear mode and were tested under an applied external magnetic field perpendicular to the position of the MRE with the varying magnetic flux densities of 0 to 800 mT. The temperature was kept constant at 25 °C during the testing, controlled by the Viscotherm VT2 (Anton Paar, Graz, Austria) that was equipped with the rheometer. The rheological tests were performed with different input parameters such that the strain amplitude sweep test was conducted with a constant frequency of 1 Hz and the strain amplitudes were varied from 0.001 to 10%. Then, the frequency sweep test was conducted from 0.1 to 10 Hz with the constant strain amplitude that was determined from the strain amplitude sweep test, which corresponded to the linear behavior of MREs. Meanwhile, the magnetic field sweep test was carried out as it is crucial for evaluating the performance of MRE towards magnetic field manipulation. The magnetic sweep test was conducted from 0 to 0.6 T.

## 3. Results and Discussion

### 3.1. Effect of Strain Sweep Test

**Figure 6:**
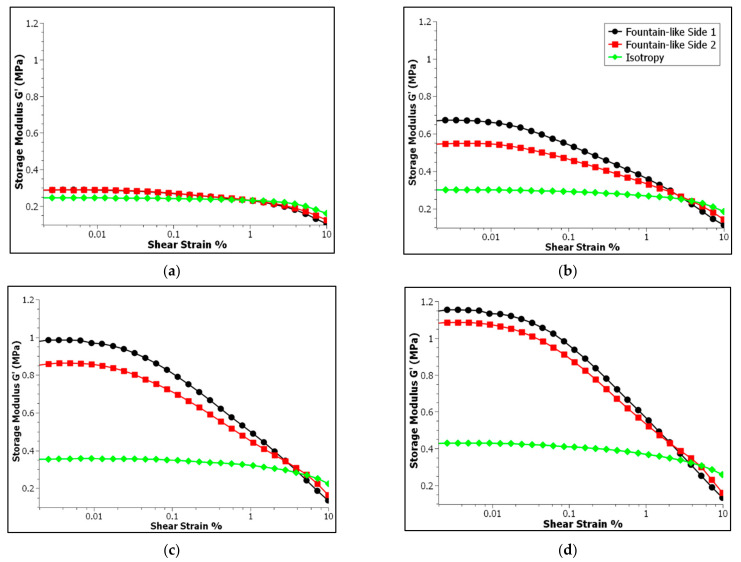
Storage modulus of MRE samples as a function of oscillation shear strains: (**a**) at 0 A, (**b**) at 1A, (**c**) at 2 A and (**d**) at 3 A, which correspond to 0, 0.2, 0.4 and 0.6 T magnetic fields, respectively.

The strain amplitude sweep test was performed in order to explore the effect of shear strains on the dynamic viscoelastic modulus of the MREs. The test was carried out by varying the amplitude strains from 0.01 to 10%, with a constant frequency of 1 Hz, and at room temperature of 25 °C. Figure 6 shows the storage modulus versus shear strain for the isotropic MRE and fountain-like MRE of Side-1 and Side-2. It is known that the MRE Side-2 has the opposite particle-chain alignment of MRE Side-1. Each of the MRE samples was tested under different magnetic fields, particularly 0, 0.2, 0.4 and 0.6 T for Figure 6a–d, respectively. In brief, all the MRE samples exhibited a strain-dependent behaviour, in which the shear storage modulus would decrease with the increase of strain amplitudes, at a specific constant magnetic field.

In Figure 6a, the fountain-like MRE samples and isotropic MRE were analysed in the off-state condition, in which no magnetic field was applied during the testing. It is observed that all the MRE samples exhibited a long horizontal straight line that represents a constant storage modulus up to 0.1% strain, before a slight downturn indicating the non-linearity of storage modulus or dependency of storage modulus of MREs at higher applied shear strain. In fact, the constant storage modulus represents a linear region of the MRE upon strain amplitudes in which the structure of the MREs has not been disrupted by the applied shear strains. It would be returned to its original state after the removal of the applied stress. However, the fountain-like MRE samples exhibited a slightly higher storage modulus, about 0.29 MPa more than the isotopic MRE (0.25 MPa), attributed to the chain-like structure of the CIPs in the MRE, particularly for both sides (Side-1 and Side-2), indicating the stiffer MRE samples compared to the isotropic MRE. Based on the previous studies, the aligned CIPs in an MRE at a specific angle would be denoted as anisotropic MRE, and it is normally stiffer than the isotropic-typed MREs [14,38,39]. Thus, the current finding is in good agreement with the previous ones like the fountain-like MREs for Side-1 and Side-2 that have the aligned CIPs at various angles possessing higher storage modulus, indicating the capability of storing more deformation energy in a linear region when subjected to the applied shear stress. For instance, Khairi et al. [14] investigated the anisotropic MRE with 0° particle’s alignment and found that it possessed the storage modulus of 0.40 MPa, which is greater than the isotropic MRE that has the storage modulus of 0.22 MPa, in a zero magnetic field condition.

On the other hand, under the on-state conditions from 0.2 to 0.6 T, particularly in Figure 6b–d respectively, the fountain-like MRE samples show a significant increment in storage modulus as compared to the isotropic MRE. In fact, the response of the storage modulus for Side-1 came out higher than the Side-2, suggesting that the influence of the particle alignments towards the magnetic field is stronger on Side-1 compared to Side-2. Initially, both sides have an identical storage modulus as shown in Figure 6a. However, with applying magnetic fields and an increase from 0.2 to 0.6 T, both fountain-like MREs became stiffer. For instance, at 0.2 T, the storage modulus of Side-1 and Side-2 are 0.67 and 0.55 MPa, respectively, compared to 0.29 MPa for both sides in the off-state condition. The values are then further increased with the increment of applied magnetic fields at 0.4 and 0.6 T. Nevertheless, the difference in storage modulus between both sides of fountain-like MREs, at a specific magnetic field, becomes lower, indicating that the MRE samples are said to approach its saturation value of storage modulus with increasing magnetic fields, as indicated in Figure 6b–d. This resultant behavior also demonstrated the influence of magnetic field strength towards the CIP–CIP interaction in the MRE samples.

Interaction of magnetic particles towards the applied magnetic field has resulted in a substantial increase in the storage modulus of the MREs. The magnetized magnetic particles would tend to align following the lines of magnetic field, and the tendency of the CIPs to align and to attract among each other via magnetic forces would create the interaction network that strengthens the MRE’s structure. This phenomenon would then make the MRE become stiffer. The higher the magnetic field strength, the stronger the magnetic forces as well as the interaction between the CIPs, thus enhancing the storage modulus of the MREs. On the other hand, the interaction between the CIPs by induced magnetic field is limited by the saturation threshold. Previous study has indicated that the magnetic field has enhanced the storage modulus of MRE to some extent before the saturation occurs [40]. Similarly, the behavior of the fountain-like MRE samples for both Side-1 and -2 that approach the storage modulus of one another have indicated that the CIP’s interaction is approaching the saturation threshold. Further increased magnetic field strength beyond the saturation value would not give a significant effect on the storage modulus of the samples. In addition, the strength of particle–particle interaction also depends on the distance between the CIPs [19,41,42,43]. The magnetic interaction between the particles decreases as the distance between them increases. In contrast to the fountain-like MRE samples, the isotropic MRE has evenly dispersed CIPs and no particle-chains are formed, resulting in a wider space between the CIPs. Consequently, the particle–particle interaction is quite lower as compared to the fountain-like MRE samples that have the aligned CIPs [41]. This behavior is also in line with the slight increment in storage modulus of the isotropic MRE as the magnetic field increased, indicating weaker interaction between the magnetized CIPs–CIPs, compared to the fountain-like MREs.

In projecting the linear viscoelastic behavior of MREs during the testing, the region called linear viscoelastic (LVE) is determined. This range implies that the molecular structure of the MRE would be returned to its original state after removal of the applied shear stress. As stated, the isotropic MRE exhibits the longest LVE region among other samples whether in the off- or on-state conditions, which is around a 3.37% strain. Meanwhile, both fountain-like MRE samples for both Side-1 and -2 possessed the LVE value at 0.19%. This finding is respective to the off-state condition as shown in Figure 6a. The LVE region between the isotropic and fountain-like MRE samples has a noticeable difference due to the stiffer fountain-like MREs attributed to the structure of the aligned CIPs in the elastomeric matrix (anisotropic). Thus, the structure is slightly rigid compared to the isotropic-typed MRE [44]. In contrast, under the influence of magnetic fields, as shown in Figure 6b–d, the LVE region for fountain-like MREs, particularly Side-1 and Side-2, becomes much shorter than the isotropic MRE, and there is a sudden downturn in both fountain-like samples. The capability to store more energy upon deformation increased with the magnetic fields; however, the MREs become stiffer and the structure phase becomes more brittle, which would lead to disruption of the structure with higher applied shear strains. Therefore, the LVE region becomes shorter with stiffer MRE. Since the LVE region would reflect the stiffness and elasticity of the MRE samples, the shorter and abrupt downturn of the storage modulus would suggest the brittle phase structure and lesser elasticity of the MRE but enhanced storage modulus or stiffness of the sample [45]. It has more capability to store deformation energy before rupture of the structure would take place.

**Figure 7:**
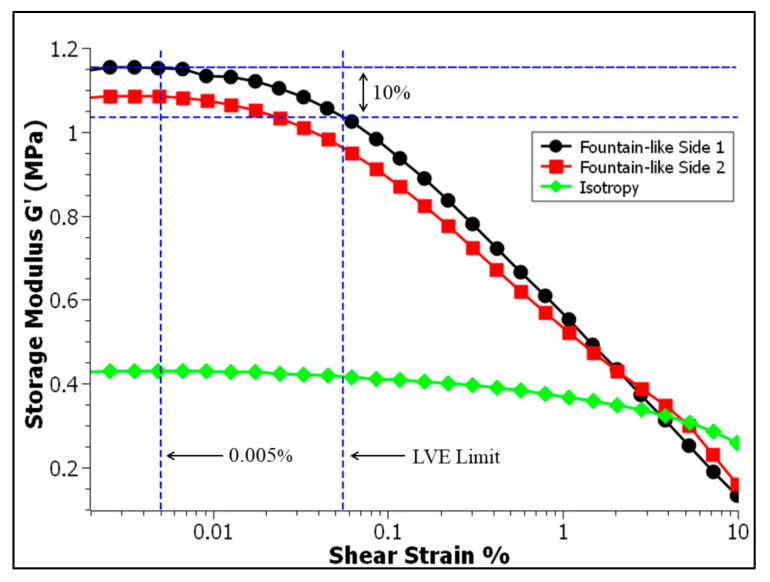
Determination of the LVE region of the MRE.

Figure 7 shows the approach of the determination LVE region, which is outlined as the function of storage modulus against the shear strains, particularly at the 0.6 T. A single LVE limit of all MRE samples would be determined for further use of other tests such as frequency sweep and magnetic field sweep tests that require a set of constant strain [14,45]. In this study, the LVE limit was determined based on the finding of fountain-like Side-1 MRE since it has the shortest LVE region, as it would cater to the LVE region of other MRE samples as well. Based on Figure 7, the LVE limit is determined based on the 10% downturn pattern from the plateau of the storage modulus [45] and respective to the projection line; the selected constant shear strain is around 0.005%. The selected value for set constant strain, however, is still under the range of LVE limit for all conditions. This ensures that the sample will revert to its original state upon deformation during any test.

### 3.2. Effect of Frequency Sweep Test

**Figure 8:**
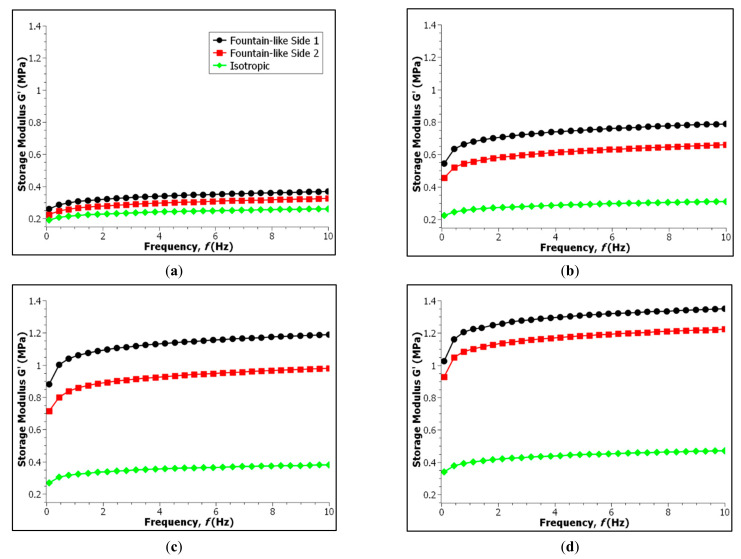
Storage modulus of MRE samples as a function of excitation frequencies: (**a**) at the off-state (0 T), (**b**) 0.2 T, (**c**) 0.4 T and (**d**) 0.6 T of applied magnetic fields.

The storage modulus for all MRE samples have been measured as a function of frequency as shown in Figure 8, at different values of magnetic fields (0, 0.2, 0.4 and 0.6 T). As seen in the figures, the storage modulus of all MRE samples have been gradually increased with increasing driving frequency, under any constant magnetic field. Figure 8a shows the increased storage modulus when the frequency was gradually increased up to 10 Hz for all three samples, particularly in the off-state condition. In addition, the fountain-like Side-1 MRE has the highest storage modulus compared to Side-2 and followed by the isotropic MRE, particularly 0.36, 0.32 and 0.26 MPa, respectively. The findings have corresponded to different stiffnesses of each MRE as it is or behavior of the samples in the absence of the applied magnetic field. Nevertheless, with presence of magnetic fields as shown in Figure 8b, particularly at 0.2 T, a significant increase in storage modulus for the fountain-like MRE samples has been observed as compared to the isotropic MRE. As discussed previously, the abrupt increment in the storage modulus is due to the fountain-like MRE samples that have chain-like structures of CIPs that normally caused stiffer MRE, which resulted in higher storage modulus. In fact, the fountain-like MRE Side-1 is stiffer compared to the MRE Side-2, although both presented the aligned CIPs in the MREs. Figure 8c,d also showed similar behaviors of the increment storage modulus of MREs with further increase in magnetic fields, which is led by the fountain-like Side-1, followed by the fountain-like Side-2 and the isotropic MRE, indicating the stiffened samples attributed to the stronger magnetic forces between the CIPs towards the applied field. The data of the storage modulus for all samples are tabulated in Table 4.

As stated, the fountain-like MRE Side-1 exhibited higher storage modulus than the MRE Side-2 and the arrangement of CIPs in both MRE samples contributed to such difference in the storage modulus. It is noted that the CIP’s alignment in Side-2 is only opposite to Side-1. In the off-state condition, the storage modulus of both sides of the fountain-like MREs shows a significant difference in the storage modulus, which is about 0.044 MPa. Then, at 0.2 T, the difference in storage modulus increased to 0.131 MPa, and the difference continues to increase at 0.4 T, with 0.210 MPa. However, at 0.6 T, the difference dropped to 0.131 MPa, denoting that both sides of fountain-like MRE are approaching its saturation with higher induced magnetic fields. In fact, a similar phenomenon has been addressed by Boczkowska et al., who stated that MRE with 0° of particle chain alignment exhibited the increment in storage modulus with applied magnetic fields, particularly from 100 to 400 mT towards the frequency variations. However, the behaviour of the storage modulus of MRE was similar to the one at 400 mT when the magnetic field was increased to 600 mT, indicating the saturation stage of the MRE with further increased induced magnetic fields [22]. 

Nevertheless, the changes in the viscoelastic properties of the MREs have been influenced by the particle chain alignments as the highest storage modulus of the MRE was achieved at 30° particle chain alignment, with 0.5 MPa in the on-state condition [22]. Meanwhile, Hapipi et al. [46], who fabricated MRE with 0° of particle chain alignment has achieved around 1.1 MPa of the storage modulus, particularly in the on-state condition with 10 Hz of an applied frequency test. Furthermore, a similar result by Khairi et al. [14], who also achieved the storage modulus by approximately 1.1 MPa, for MRE with 0° particle chain alignment. In comparison to the current MRE, fountain-like alignment of CIPs, however, has shown a slight improvement in the storage modulus, around 1.3 MPa respective to the on-state condition and with 10 Hz of a frequency sweep test.

### 3.3. Effect of the Magnetic Field Sweep Test

**Figure 9:**
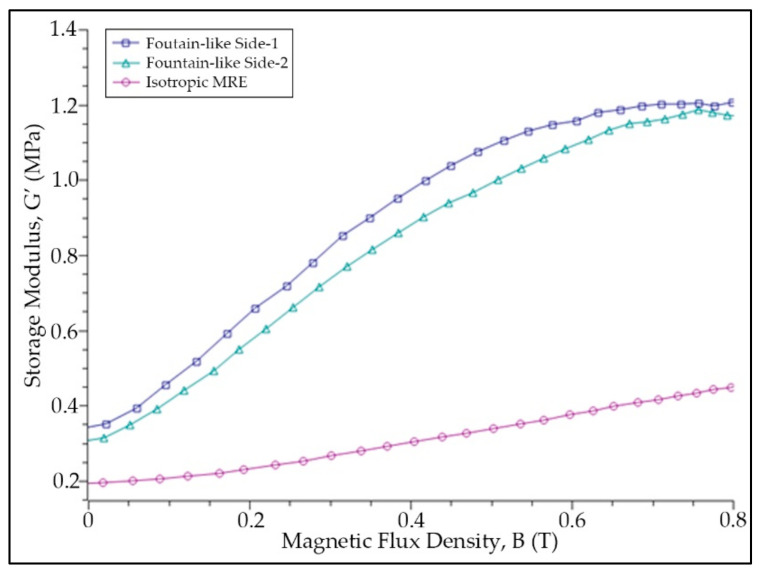
Storage modulus of MRE samples as a function of magnetic field sweep.

**Figure 10:**
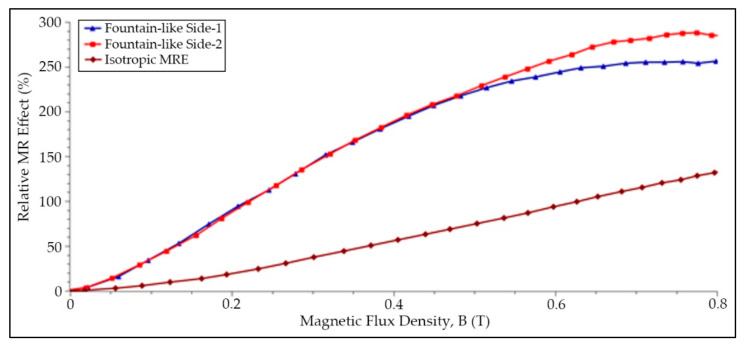
Relative MR Effect of each MRE sample.

The relationship between the storage modulus of MRE samples at different magnetic field values was measured and presented in Figure 9, while Figure 10 represents the relative MR effect of MRE samples that projected from Figure 9. As observed, all the MRE samples exhibit a similar pattern as the shear storage modulus increased with the increasing magnetic fields. The MREs are said to become stiffer with magnetic fields. This is a typical phenomenon in MRE in which, when a magnetic field is applied to the MRE, the magnetic dipole moment of the CIPs within the matrix is induced. Hence, more energy is required to break the magnetic forces between the particles, causing the shear modulus of MRE to change and enhance [11]. As presented in Figure 9, the storage modulus for fountain-like Side-1 MRE was slightly higher than the fountain-like Side-2, followed by the isotropic MRE. Since the fountain-like MRE Side-1 has a stronger interaction between the particles, the elastomer composite appears to be stiffened more than the Side-2, even at the initial state. Nonetheless, both storage modulus of fountain-like Side-1 and Side-2 MREs increased almost at the same rate, up to 0.5 T as indicated in Figure 9 and Figure 10. Then, with a further increase in a magnetic field up to 0.8 T, the increment behavior in the storage modulus of both fountain-like MREs shows a different trend. In particular, the Side-2 MRE demonstrates a further increase in storage modulus, which is contradictory to the Side-1 MRE that shows a fading increment in storage modulus, indicating the saturation state of the Side-1 MRE with higher applied magnetic fields. In other words, the fountain-like Side-1 MRE has achieved its saturation stage of storage modulus quicker than the Side-2 MRE. Subsequently, both lines of storage modulus are approaching one another, as presented in Figure 9. In general, at higher values of magnetic fields, particularly more than 0.8 T, the CIPs would achieve its maximum magnetization; therefore, a further increase in the magnetic field values might not affect the improvement in the properties of MRE. 

Table 5 summarizes the zero-field modulus, maximum storage modulus at 0.8 T, magneto-induced modulus as well as a relative MR effect of the evaluated MRE samples. Magneto-induced modulus is also known as the absolute MR effect, and it is expressed as in Equation (1), while Equation (2) describes the relative MR effect:

(1) $$\Delta G={G^{\prime }}_{max}-G_{0}$$

(2) $$MR_{\mathrm{effect}}=\frac{\Delta G}{G\prime_{0}}\times 100\%$$

where *G’_max_* is the storage modulus at magnetic saturation, and *G_0_* is the storage modulus at a zero magnetic field. Based on Table 5 and projected data of a relative MR effect of MREs in Figure 10, it is observed that the fountain-like Side-2 MRE exhibits a greater MR effect compared to Side-1 MRE attributed to the lower initial storage modulus, and *G_0_*, particularly 0.29 MPa, compared to Side-1 MRE (0.35 MPa), which is stiffer at the initial state. The storage modulus at the highest applied magnetic field, particularly 0.8 T, however, reveals that the fountain-like Side-1 and Side-2 MREs have almost the same values, contributing to the significant difference in the absolute and relative MR effect of Side-2 higher than the Side-1. In fact, as discussed in the previous paragraph, the Side-1 MRE possessed the fading increment in a storage modulus with magnetic fields, resulting in a lower MR effect of Side-1 MRE (225%) as compared to Side-2 MRE with 289%. In comparison with the previous study, Hapipi et al. fabricated the anisotropic MRE at 0° of particle chain alignment with 70 wt.% of CIPs, and the study achieved about 125% of the relative MR effect [46]. Meanwhile, another study by Khairi et al. who fabricated the anisotropic MRE at 0° particle chain alignment, with also 70 wt.% of CIPs content, has achieved about 145% of the relative MR effect [14]. Apart from that, the study by Tian et al. [32] showed that the MRE with 45° of particle’s chain alignment has exhibited about 35% of the relative MR effect, particularly in the shear direction that compressed the 45° particle–chain arrangement in the MRE. In addition, Yao et al. who fabricated MRE with 33 wt.% of CIPs at also 45° of particle chain alignment has possessed about 0.55 MPa of magneto-induced modulus that resulted in around 78% of the respective MR effect [13]. On the other hand, Zhang et al. stated that MRE with 50 wt.% of CIPs and fabricated at 15° of the CIP’s alignment has the relative MR effect of about 75%. However, their study focused on the PDMS-based MRE [34]. Nevertheless, the current study that fabricated MRE with a fountain-like shape of particle chain alignment has presented higher values of relative MR effect as compared to those of previous studies. It is expected that the fountain-like arrangement of CIPs in the MRE has a stronger magnetic interaction between the particles and magnetic fields, simultaneously towards the applied shear stress. It is noted that these studies were free from additives that mainly focused on the effect of various degrees of CIP arrangement in an MRE and those of related studies have been summarized in Table 6.

### 3.4. Morphological Characterization and Analysis

**Figure 11:**
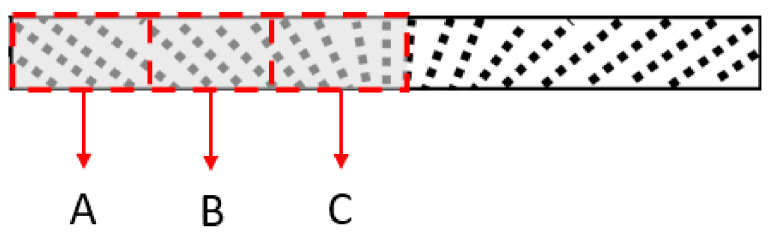
The illustration of Regions A, B and C of cross-section MRE sample under morphological analysis.

**Figure 12:**
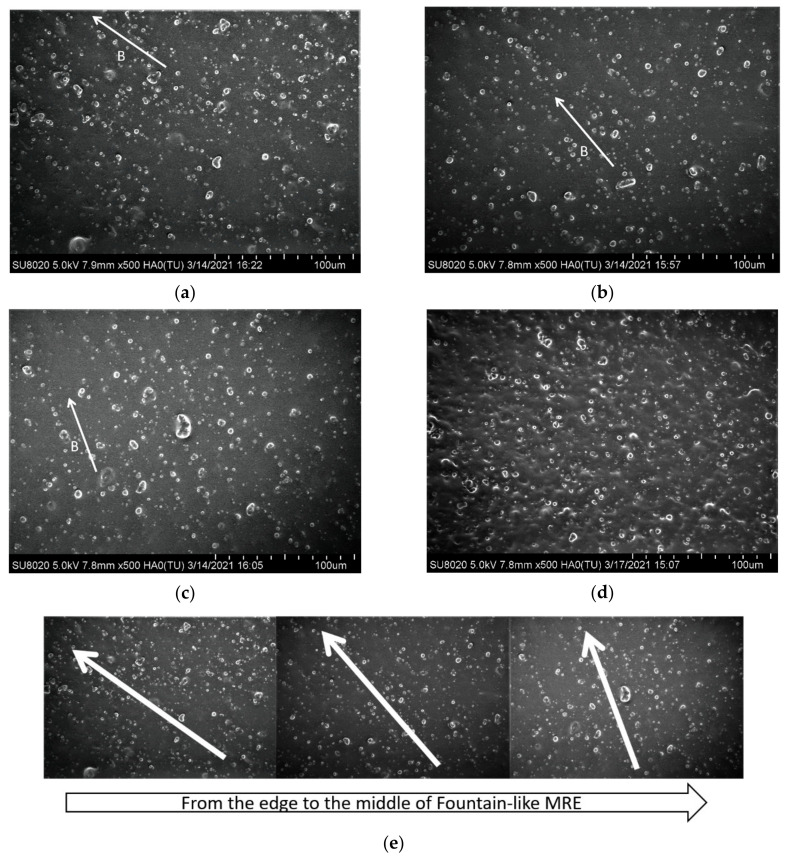
Morphologies of fountain-like MRE corresponded to (**a**) region A, (**b**) region B, (**c**) region C and (**d**) isotropic MRE. (**e**) shows the combined FESEM images from regions A, B and C. The direction of the magnetic field is shown by a white arrow and is labelled as B.

Figure 11 illustrates the cross-section of the fountain-like MRE sample that is divided into three regions, denoted as A, B and C. The regions represent the FESEM observation that focused on the formation of fountain-like particles chains, from the edge to the middle region. Meanwhile, the FESEM images of the fountain-like MRE are shown in Figure 12, corresponding to regions A, B and C, and in comparison to the isotropic MRE.

Figure 12a–c present the surface morphologies of fountain-like MRE; particularly, Side-1 corresponded to regions A, B and C, respectively, as indicated in Figure 11. Meanwhile, Figure 12d shows the isotropic MRE and Figure 12e is the combined images of Figure 12a–c. It can be observed that, from edge (a) to the center of the sample (b), the particle chains of the CIPs showed the changing angles, approaching 0° to the *y*-axis, indicating the fountain-like shape of the particle’s arrangement in the MRE. The distribution and arrangement of CIPs also correspond to the magnetic field directions as controlled by the designed mold and predicted via FEMM analysis, as discussed in Section 2.2. The interaction and attraction among the CIPs caused them to align following the flow of magnetic fields when it was applied to the MRE melt during the curing process. In the curing device, the utilization of permanent magnets in the curing apparatus has an impact on the CIP chains’ formation since it acts as a pick-up magnetic field that caused the formation of fountain-like lines of magnetic flux density in the curing mold. Meanwhile, around 0.2 T of applied magnetic field has guided the CIPs to align along the curved magnetic flux lines, resulting in the fountain-like shape of the CIP alignment. The CIPs got fixed and trapped in the elastomeric matrix in the form of fountain-like chains upon the curing process, producing Fountain-like Side-1 MRE. On the other hand, the isotropic MRE as in Figure 12d shows the scattered CIPs as the melt MRE was cured without the presence of the magnetic field, leading to randomness locked CIPs in the matrix phase. 

**Figure 13:**
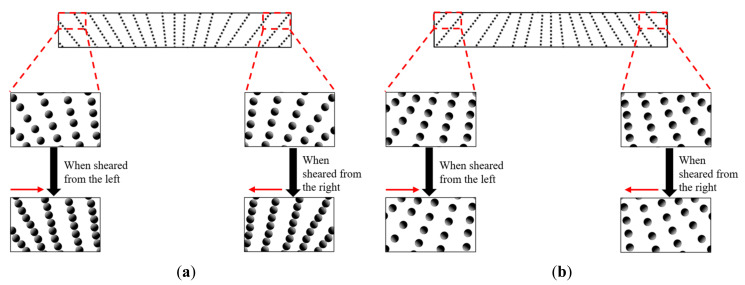
Schematic diagram of fountain-like MREs respective to the applied shear stress: (**a**) Fountain-like Side-1, (**b**) Fountain-like Side-2. The magnified square area represents the particle chains at edges of the MREs.

It is well known that the particle distribution is one of the important parameters that led to the changes in the rheological properties of MRE, particularly on the resultant stiffness and MR effect. Due to fountain-like alignment of CIPs in the MREs, some changes of the particle–matrix interactions during the shearing force were expected to occur as some particle’s arrangement might be expanded and some were compressed, respective to the direction of applied shear stress. The schematic diagram of the fountain-like MREs and the CIPs behavior during the applied shear stress are depicted in Figure 13. In particular, Figure 13a demonstrates the changes of particle chains when the Fountain-like Side-1 MRE was sheared oscillatory, from left to right and vice-versa. Either way, the shear deformation has compressed the particle chains as the CIPs that were arranged in the fountain-like shape looked as if they were opposing the applied shear stress. Accordingly, the gap between the particles were observed to be reduced, and the compressed CIPs have led the MRE to slightly resist deformation, thus enhancing the stiffness of the MRE. The effect also caused the shear stress to be increased, simultaneously increasing the storage modulus of the MRE. The similar result has been explained by Zhang et al. [34] who stated in their theoretical modelling that, when the average distance between the particles was reduced, the shear stress of the material would be increased. On the contrary, the shear stress that was applied onto the Fountain-like Side-2 MRE would cause the particle chains to be expanded as the CIPs that were arranged in such manner would have less opposition to the applied shear stress. The effect also caused the gap between the CIPs to increase, causing the MRE to deform slightly easier, as indicated in Figure 13b. As a result, the fountain-like Side-2 MRE performed slightly lower storage modulus as compared to fountain-like Side-1 MRE, as has been discussed in previous sections. Anyhow, both fountain-like MRE samples exhibited higher storage modulus as compared to the isotropic MRE as the aligned CIPs have possessed a closer gap between the particles and stronger magnetic forces, especially when there is the presence of magnetic fields. This phenomenon has driven the stiffness of MREs to be increased accordingly and perhaps the proposed fountain-like CIPs in the MRE would cater to shear stress in both directions, which resulted in improved distribution viscoelastic properties of MRE, on both sides during the applied stress.

## 4. Conclusions

This work focused on the fountain-like alignments of CIPs in an MRE and their effect on the resultant storage modulus and MR effect, in comparison to the isotropic MRE. The development of the fountain-like curing apparatus has driven the CIPs to be arranged in fountain-like structure in order to configure various angles of CIP’s alignment in the MRE. Around 0.2 T of magnetic field strength was induced to generate fountain-like lines of magnetic flux density with the help of the permanent magnet on the curing device that acted as pick-up magnetic flux lines. The arrangement of fountain-like CIPs chains in the MRE can be either in the same direction with the magnetic flux lines or inverted directions, which was associated as Side-1 or Side-2, respectively. The results revealed that the applied shear direction in the oscillatory shear mode test has influenced the arrangement of the CIPs in the MREs as both fountain-like CIPs of Side-1 and Side-2 exhibited differences in storage modulus. In fact, the applied shear stress to the fountain-like Side-1 MRE caused the CIPs to be cramped, which led the MRE to resist deformation and increase in storage modulus as well as stiffness, as compared to the fountain-like Side-2 MRE that possessed the expansion of arranged CIPs. In fact, at lower magnetic fields, Side-1 and Side-2 MREs have a significant difference in the storage modulus, around 0.35 MPa and 0.29 MPa, respectively. However, both approach the similar saturated values at higher magnetic fields, around 0.79 MPa and 0.82 MPa, respectively. This behavior was supported by a magnetic field sweep test that was run for Side-1 and Side-2 MREs, leading the value of the MR effect at around 226% and 283%, respectively. Nevertheless, the fountain-like Side-2 MRE exhibits a higher MR effect, since it has a lower initial storage modulus and greater magneto-induced modulus compared to Side-1 MRE. These findings have indicated the significant influence of the fountain-like alignment of CIPs towards the performance of the MREs, especially in both directions of applied shear stress.

## Figures and Tables

**Table 1:** Illustration of the types of CIP alignment in MRE samples.

Particle Alignment	Type	Description
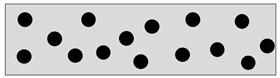	Isotropic	CIPs randomly distributed in MRE.
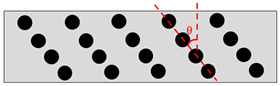	Anisotropic	CIPs aligned in one direction. Can be any angle i.e., θ = 0°, 30°, 45° or 90°.
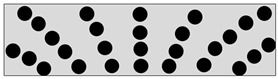	Fountain-Like	New proposed alignment: CIPs are aligned in the form of a fountain-like structure.
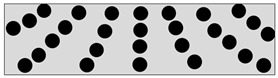	Inverted fountain-like	CIPs are aligned in the inverted form of fountain-like structure.

**Table 2:** Types of MRE samples.

Sample	Type	Description
1	Isotropic	Isotropic MRE
2	Fountain-Like Side-1	MRE with “fountain-like” particle chain alignment
3	Fountain-Like Side-2	MRE with inverted “fountain-like” particle chain alignment

**Table 3:** List of parts and materials for the fabrication of electromagnetic coil.

No.	Part List	Material
1	Bobbin	Plastic
2	Coil	Copper wire dia. 0.9 mm
3	Cover	Carbon steel, AISI 4140
4	Permanent magnet	Disc-shaped, dia. 20 mm, ht. 5 mm, B = 0.36 T
5	MRE mold	Acrylonitrile butadiene styrene

**Table 4:** Maximum storage modulus for all MRE samples and the difference in storage modulus for both sides of fountain-like MREs, in the frequency sweep test.

Magnetic Field Strength	MRE			Difference of fountain-like MREs (*G_Max_*) (MPa)
	Isotropic MRE	Fountain-Like MRE Side-1	Fountain-Like MRE Side-2	
	*G_Max_* (MPa)	*G_Max_* (MPa)	*G_Max_* (MPa)	
0 T	0.262	0.363	0.319	0.044
0.2 T	0.314	0.789	0.658	0.131
0.4 T	0.385	1.187	0.977	0.210
0.6 T	0.475	1.351	1.220	0.131

**Table 5:** The zero-field storage modulus (*G_0_*), the magnetically induced modulus (Δ*G*), and the relative MR effect of the MRE samples.

MRE Samples	*G_0_* (MPa)	*G_Max_* (MPa)	Δ*G* (MPa)	Relative MR Effect (%)
Fountain-like Side-1	0.35	1.14	0.79	225.71
Fountain-like Side-2	0.29	1.11	0.82	282.76
Isotropic	0.23	0.49	0.26	113.04

**Table 6:** Comparison of a particle’s alignment and MR effect of MREs.

No.	Matrix	Alignment	Filler	wt.% (CIPs)	Storage Modulus (MPa)	Relative MR Effect (%)	Ref.
1	Silicone rubber	0°	CIP	70	0.98	125	[46]
2	Silicone rubber	0°	CIP	70	0.98	145	[14]
3	Silicone rubber	45°	CIP	70	NA	35	[32]
4	Silicone rubber	45°	CIP	33	1.2	~79	[13]
5	PDMS	15°	CIP	50	NA	75	[34]
6	Silicone rubber	Fountain-like Side-1	CIP	70	1.14	225	Current study
		Fountain-like Side-2			1.11	289	

## Data Availability

The raw/processed data required to reproduce these findings cannot be shared at this time as the data also form part of an ongoing study. In the future, however, the raw data required to reproduce these findings will be available from the corresponding authors.
